# CircRNA75 and CircRNA72 Function as the Sponge of MicroRNA-200 to Suppress Coelomocyte Apoptosis *Via* Targeting Tollip in *Apostichopus japonicus*

**DOI:** 10.3389/fimmu.2021.770055

**Published:** 2021-11-17

**Authors:** Jiqing Liu, Xuelin Zhao, Xuemei Duan, Weiwei Zhang, Chenghua Li

**Affiliations:** ^1^ State Key Laboratory for Quality and Safety of Agro-products, Ningbo University, Ningbo, China; ^2^ Collaborative Innovation Center for Zhejiang Marine High-efficiency and Healthy Aquaculture, Ningbo University, Ningbo, China; ^3^ Laboratory for Marine Fisheries Science and Food Production Processes, Qingdao National Laboratory for Marine Science and Technology, Qingdao, China

**Keywords:** *Apostichopus japonicus*, circRNA, miR-200, Tollip, apoptosis

## Abstract

Circular RNAs (circRNAs) act as essential regulators in many biological processes, especially in mammalian immune response. Nonetheless, the functions and mechanisms of circRNAs in the invertebrate immune system are largely unclarified. In our previous work, 261 differentially expressed circRNAs potentially related to the development of *Apostichopus japonicus* skin ulceration syndrome (SUS), which is a major problem restricting the sea cucumber breeding industry, were identified by genome-wide screening. In this study, *via* miRanda analysis, both circRNA75 and circrRNA72 were shown to share the miR-200 binding site, a key microRNA in the SUS. The two circRNAs were verified to be increased significantly in LPS-exposed primary coelomocytes, similar to the results of circRNA-seq in sea cucumber under *Vibrio splendidus*-challenged conditions. A dual-luciferase assay indicated that both circRNA75 and circRNA72 could bind miR-200 *in vivo*, in which circRNA75 had four binding sites of miR-200 and only one for circRNA72. Furthermore, we found that miR-200 could bind the 3’-UTR of Toll interacting protein (Tollip) to negatively mediate the expression of Tollip. Silencing Tollip increased primary coelomocyte apoptosis. Consistently, inference of circRNA75 and circRNA72 could also downregulate Tollip expression, thereby increasing the apoptosis of primary coelomocytes, which could be blocked by miR-200 inhibitor treatment. Moreover, the rate of si-circRNA75-downregulated Tollip expression was higher than that of si-circRNA72 under an equivalent amount. CircRNA75 and circRNA72 suppressed coelomocyte apoptosis by sponging miR-200 to promote Tollip expression. The ability of circRNA to adsorb miRNA might be positively related to the number of binding sites for miRNA.

## Introduction

CircRNAs are a new type of non-coding RNA (ncRNA) with neither 5’ to 3’ polarity nor a polyadenylation tail and with covalently closed circular structure (1). With the development of high-throughput sequencing and bioinformatics, researchers have discovered more than 10,000 circRNAs in many animals, such as fruit fly (2), fish (3), mouse (4), monkey (5), and human (6). Moreover, the presence of circRNAs has also been confirmed in plants (7), fungi (8), and protists (9). Emerging studies suggested that some circRNAs played crucial roles under physiological and pathological conditions (10). Knockout experiments proved that neuronal function and development may be mediated by circRNAs (11). The onset of many diseases or worsening of tumor formation is due to abnormal circRNA expression in the internal environment (12, 13).

Three main biological functions of circRNAs have been identified, as follows: (a) they act as competing endogenous RNAs to bind microRNAs (miRNA sponges) (14), (b) act as transcription regulators (15), and (c) encode proteins with molecular weight and functions different from those of their host gene (16). Among the biological functions of circRNAs, acting as a miRNA sponge is the most frequently studied function that allows them to participate in many physiological and pathophysiological processes (17). For instance, in renal cell carcinoma (RCC), circTLK1 could adsorb miR-136-5p to upregulate CBX4 expression, giving rise to tumorigenesis and promoting RCC development (18). In osteoarthritis (OA), abnormal circRNA-UBE2G1 facilitates the proliferation and migration in the LPS-induced OA cell model through the miR-373/HIF-1a axis (19).

*Apostichopus japonicus* is one of the most crucial aquaculture species and has great economic value in China (20). Unfortunately, with the expansion of culturing scale, various viral and bacterial disease outbreaks, especially SUS caused by *Vibrio splendidus*, result in serious economic loss (21, 22). Therefore, studying the innate immune defense mechanisms is essential to prevent and treat diseases affecting the aquaculture of *A. japonicus*. Meanwhile, circRNAs, as miRNA sponges, have an important effect on the innate immune of many organisms (23). For instance, circHIPK2 could regulate autophagy and ER stress to significantly inhibit astrocyte activation *via* the targeting of miR124-2HG (24). Knockdown of circRNA cPWWP2A led to increased miR-579 activity and triggered macrophage apoptosis (25). In our previous studies, we found that 261 circRNAs were differentially expressed in the coelomocyte of SUS-affected *A. japonicas* compared with healthy ones (26). However, the mechanism of circRNA in SUS-infected *A. japonicas* is still unknown.

In this study, two immune-related circRNAs, namely, circRNA75 and circRNA72, were significantly upregulated in LPS-exposed coelomocytes. These two circRNAs functioned as the sponge of miR-200 to regulate Toll interacting protein (Tollip) expression and further modulate coelomocyte apoptosis. CircRNA75 had more binding sites showing higher sponge ability compared with circRNA72. Our present work provided new insights into the immune-regulation mechanism of *A. japonicas* under a pathogen challenge.

## Materials and Methods

### Experimental Animals and Ethics Statement

Healthy *A. japonicus* (weight 98 g ± 14 g) were purchased from the Dalian Pacific Aquaculture Company and temporarily reared in aerated seawater (salinity 28 ± 0.5; temperature 16 ± 0.5°C; pH 8 ± 0.1) for 5 days before the experiments. All experiments were performed in accordance with the advice in the Guide for the Care and Use of Laboratory Animals of the National Institutes of Health. The experimental protocols were approved by the Research Animal Ethics Committee of Ningbo University, China.

### RNA Extraction and Quantitative Real-Time PCR

The total RNA from coelomocytes was isolated with RNAiso plus (TaKaRa, Dalian, China) according to the manufacturer’s instructions. RNA concentration was measured by Nanodrop, and each paired sample was adjusted to the same concentration (1,000 ng/ul). For PCR of mRNA and circRNA, the cDNA was synthesized by the PrimeScript™ RT reagent Kit (TaKaRa, Dalian, China). For the qRT-RCR of miRNA, cDNA was synthesized using the miScript II RT Kit (Qiagen, Dusseldorf, Germany). qRT-PCR was performed using the TB Mix (TaKaRa, Dalian, China) on a 7500 real-time PCR detection system. The relative expression was calculated by the 2^−ΔΔCT^ method. Actin served as the endogenous control for circRNA and mRNA. The miRNA expression levels were normalized against RNU6B. Primer sequences are listed in **Table 1**.

**Table 1:** Primers used in this study.

Primer name	Primer sequence (5’-3’)	Used for
CircRNA75-F	TGGAGAGAAATGAAGGTTTTACA	Divergent PCR
CircRNA75-R	TCAAGGTATCCGTCTGGTTG	
CircRNA72-F	TCACATCAGTCTCCTGGTTTTG	
CircRNA72-R	CGCCTGTCTGGTAACGAAG	
Linear75-F	TTTCTATCAACAACCTGACTACCCT	Convergent PCR
Linear75-R	ATTGGATGTTATGATTACCCCG	
Linear72-F	CAACACCAGAGGAAAAGCCCAGAAA	
Linear72-R	ACGGGGTCAGGTCTGTCACGGTAAA	
Ajβ-actin-F	CCATTCAACCCTAAAGCCAACA	Real-time PCR
Ajβ-actin-R	ACACACCGTCTCCTGAGTCCAT	
AjTollip-F	GATAAACCACGATGAGCAAACAGC	
AjTollip-R	CTTGGGTTCTTGGCTCCATTCATA	
MiR-200	UAAUACUGUCUGGUGAUGAUGUU	
RNU6B	CGTGAAGCGTTCCATATTTTAA	
Universe miRNA primer	Trans miScript universal primer	
Luc-circRNA75-1F	CCCTCGAGCATCGTGCTTCCTTTCTTGG	Vector construct
Luc-circRNA75-1R	GGGTTTAAACTTGATTGGTGCTCTGCGTAA	
Luc-circRNA75-2F	CCCTCGAGTCAAGGAAACCTACTAACTGAAAA	
Luc-circRNA75-2R	GGGTTTAAACACTGGTGACCTATTGGCTTC	
Luc-circRNA75-3F	CCCTCGAGGGACCTTATTGATAGGCACC	
Luc-circRNA75-3R	GGGTTTAAACACAAATCTAACAGAAACCTTCG	
Luc-circRNA75-4F	CCCTCGAGTTTACCTATACAGAAGAACATCGT	
Luc-circRNA75-4R	GGGTTTAAACTTTTGATTATTTTGCGAAGG	
Luc-circRNA72-1F	CCCTCGAGAGTGGCAGCCTGGATTCATTT	
Luc-circRNA72-1R	GGGTTTAAACGTCCCGTACCCTGATTCTTTC	
Luc-circRNA72-2F	CCCTCGAGCTTTCATTACTTTCCCAACACTG	
Luc-circRNA72-1R	GGGTTTAAACGGAGGAACCTTACTGCCATC	
Tollip-3’UTR-F	CCCTCGAGTGAACAATCATTTTTATTCCCCACT	
Tollip-3’UTR-R	GGGTTTAAACCGAGATAACCACTGAGTGCTGA	
MyD88-3’UTR-F	CCCTCGAGAAAGAACCAAATGAGTTGAACTG	
MyD88-3’UTR-R	GGGTTTAAACTTTCTATTTACTTTCATCAAGCA	
TRAF6-3’UTR-F	CCCTCGAGTTAGTTGGTCCTGTGTTGATGC	
TRAF6-3’UTR-R	GGGTTTAAACTTGTTTTCATTGTTCTCCTCCA	
P105-3’UTR-F	CCCTCGAGCTGTTCAGTTGGTCTTGG	
P105-3’UTR-R	GGGTTTAAACCGTATTTCACTTGTTTTATAGTA	
Negative control	UUCUCCGAACGUGUCACGUTT	Negative control for siRNA interference
	ACGUGACACGUUCGGAGAATT	
CircRNA75 siRNA	GAAGUACUAAUUCAGAUUCTT	CircRNA silencing
	GAAUCUGAAUUAGUACUUCTT	
CircRNA72 siRNA	GACGUGGGGGUGGAAGAAUTT	
	AUUCUUCCACCCCCACGUCTT	
Tollip siRNA	GGAUGAUCAAUCUAGUCUUTT	
	AAGACUAGAUUGAUCAUCCTT	
MiR-200 mimics	UAAUACUGUCUGGUGAUGAUGUU	MiR-200 overexpression
	CAUCAUCACCAGACAGUAUUA UU	
Mimics negative control	UUCUCCGAACGUGUCACGUTT	
	ACGUGACACGUUCGGAGAATT	
MiR-200 inhibitor	AACAUCAUCACCAGACAGUAUUA	MiR-200 silencing
Inhibitor negative control	CAGUACUUUUGUGUAGUACAA	

### Western Blot Analysis

The total protein was extracted from coelomocytes by cell lysis buffer (Beyotime, Shanghai, China) and quantified at 50 μg/ml with the BCA protein assay kit (Cwbio, Jiangsu, China). The protein was separated by 12% SDS-PAGE gels and transferred onto a PVDF membrane by wet blotting. The membranes were blocked with 5% non-fat powdered milk at room temperature for 2 h and incubated with a primary antibody (1:500 diluted in 1% BSA) at 4°C for 8 h. Then, they were incubated with a secondary antibody (1:10,000 diluted in 1% BSA) at room temperature for 1.5 h. Finally, the blots were measured by Western ECL Substrate (Bio-Rad, California, USA), and images were obtained by using an Omega Lum C imaging system (Aplegen, California, USA).

### RNase R Resistance Analysis of circRNAs

Coelomocyte RNA (1 μg) was mixed with 1 U/μg Ribonuclease R (RNase R; Epicenter, WI, USA) or left unmixed at room temperature for 30 min. The incubated RNAs were reverse-transcribed to cDNA with specific primers for the RT-PCR assay.

### LPS-Exposed Primary Coelomocytes Culture

The protocol used for culturing primary coelomocytes was the same as that used in our previous work (27). The coelomic fluids from healthy sea cucumber were passed through a 300-mesh filter to remove large tissue debris. Then, the coelomic fluids were mixed well with an equal volume of anticoagulant solution (0.02 M EGTA, 0.48 M NaCl, 0.019 M KCl, 0.068 M Tris–HCl, pH = 7.6) and centrifuged at 300×*g* at 4°C for 10 min to remove the supernatant. After resuspension and centrifugation twice with isotonic buffer (0.001 M EGTA, 0.53 M NaCl, 0.01 M Tri–HCl, pH = 7.6), the coelomocytes were mixed well with L-15 cell medium (Sangon, Shanghai, China) containing penicillin (100 U ml^−1^) and streptomycin sulfate (100 mg ml^−1^) to obtain a final concentration of 10^6^ cells. The mixture was added to a 6-well culture plate at a volume of 2 ml per well and cultured at 16°C overnight. Then, the coelomocytes were challenged with 1 μg ml^−1^ of LPS (Sigma, California, USA) for 0, 1, 3, 6, 12, and 24 h. Three biological replicate cells comprised a group for the extracted RNA and protein.

### Vectors Construction and Cell Transfection

For luciferase reporter plasmids, the binding site sequences of circRNA75, circRNA72, target genes, and their mutant sequences were inserted into the psiCHECK-2 plasmids (Promega, Madison, USA). The mutant sequences were obtained by the Fast Mutagenesis System (Trans, Beijing, China). SiRNA (which covered the back-splicing region of circRNAs for silencing), miR-200 mimics, and miR-200 inhibitor were synthesized from GenePharma (Shanghai, China), and the sequences are listed in **Table 1**. Both plasmids and oligonucleotides were transfected into the EPC cell lines (the epithelioma papulosum cyprinid) and primary coelomocytes using Lipofectamine 6000 (Beyotime, Shanghai, China) according to the manufacturer’s protocols. In brief, 1 ul of siRNA, miRNA mimics, or miRNA inhibitor (20 mM) was mixed with 2 ul of Lipofectamine 6000 and incubated at room temperature for 15 min. Then, the 3 ul transfection solutions were added to each well of a 24-well plate.

### Dual-Luciferase Assay

The recombinant plasmids (200 ng per well), miR-200 mimics NC, and miR-200 mimics (200 nM) were co-transfected into EPC cells. After transfection for 48 h, the culture medium was removed, and each well was washed twice with 100 ul of PBS. Next, each well was treated with 20 μl of 1× Passive Lysis Buffer (PLB) for 25 min at room temperature to dissolve cells. Then, 100 μl of Luciferase Assay Reagent II (LAR II) was added to each well, and the Firefly luciferase activity was immediately measured by a GloMax 96, 20/20 luminometer (Promega, Madison, USA). After measuring the Firefly luciferase activity, 100 ul of Stop & Glo^®^ Reagent was added accordingly to measure the *Renilla* luciferase activity. All group data came from six biological replicates. One-way (ANOVA) was used to analyze the significant differences between control and experimental groups.

### Cell Apoptotic Assay

For the apoptotic assay, the primary coelomocytes were seeded into a 24-well plate for 8 h. After transfection of siRNA, miR-200 mimics, and miR-200 inhibitor for 48 h, the coelomocytes were harvested by centrifugation at 300×*g* for 10 min. The apoptosis levels of coelomocytes were determined by the Annexin V-FITC apoptosis detection kit (Beyotime, Shanghai, China) according to the manufacturer’s instructions. In short, 10^6^ coelomocytes were incubated in 200 μl of Annexin V-FITC binding buffer containing 10 μl of PI and 5 μl of Annexin V-FITC to stain, which were left to stand in the dark at 25°C for 10 min. Finally, the apoptosis levels were detected using Gallios (Beckman, California, USA).

## Results

### Identification of Two CircRNAs Related to SUS Development

**Figure 1:**
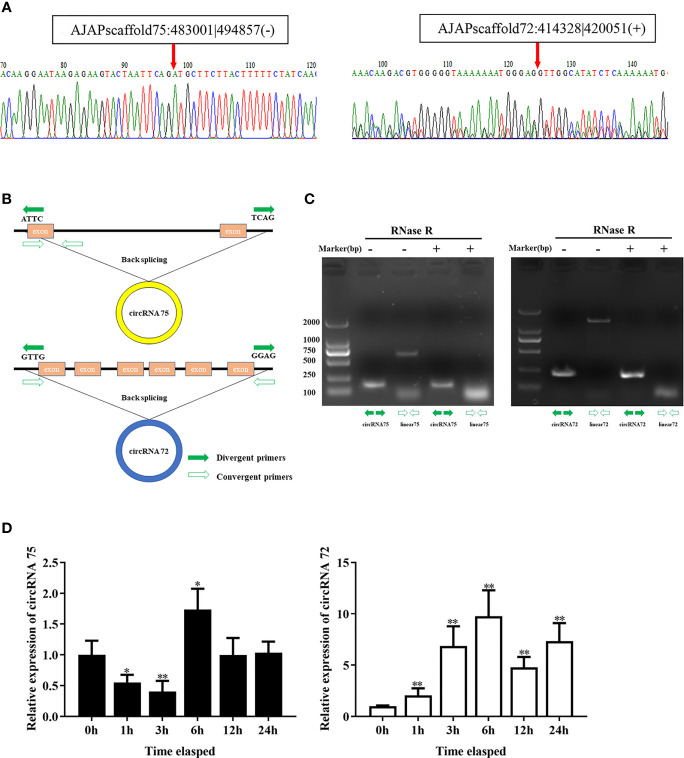
Identification of the two circRNAs related to SUS development. **(A)** The back-splicing junctions of circRNA75 and circRNA72 were verified by RT-PCR and Sanger sequencing, respectively. **(B)** Schematic illustration exhibited the formation of the two circRNAs through the circularization of their host genes. **(C)** Relative expressions of circRNAs and linear RNA in coelomocytes were detected by RT-PCR upon RNase R treatment. **(D)** The expression patterns of circRNA75 and circRNA72 under LPS stimulation. *p < 0.05 and **p < 0.01.

In our previous study, we found that miR-200 was abundant in coelomocytes of SUS and might play important roles in the immune response of *A. japonicus* (28). On the basis of the result, we screened two circRNAs (circRNA75 and circRNA72) among 261 circRNAs that exhibited miR-200 potential binding sites. To assure the circle structure of circRNA75 and circRNA72, reverse transcription PCR with divergent primers and Sanger sequencing were performed to verify their existence and the splicing junctions of the two circRNAs (**Figure 1A**). Genomic annotation of *A. japonicus* by back-splicing showed that circRNA75 and circRNA72 came from one exon of fibrinogen-like protein A and six exons of HSP97, respectively (**Figure 1B**). To further confirm the existence of circRNA75 and circRNA72, we treated total RNA of coelomocytes with RNase R and found that the linear RNAs (the corresponding linear RNAs of circRNA75 and circRNA72) were digested by RNase R rather than circRNA75 and circRNA72, which were resistant to RNase R digestion (**Figure 1C**).

To further address the immune function of the two circRNAs, the expressions of circRNA75 and circRNA72 in LPS-exposed coelomocytes were detected by qRT-PCR (**Figure 1D**). The results showed that circRNA75 and circRNA72 transcripts were significantly upregulated with a 1.73- and 7.18-fold increase at 6 h (*p <*0.01), respectively. These results were consistent with the results of the circRNA-seq differential expression analysis.

### CircRNA75 and CircRNA72 Could Bind to MiR-200 by Dual-Luciferase Reporter Assay

**Figure 2:**
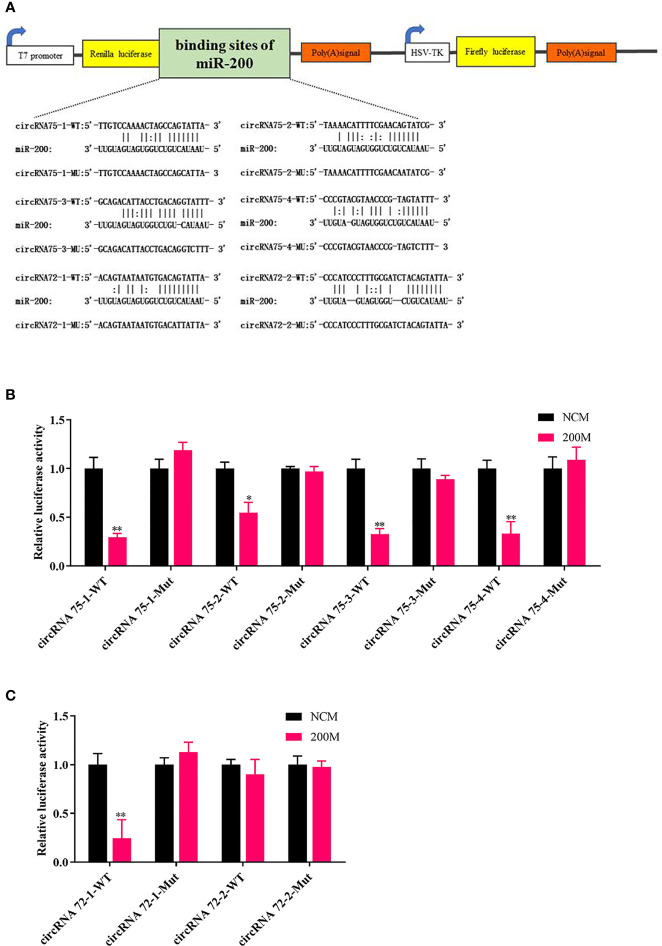
CircRNA75 and circRNA72 could bind to miR-200 as seen in the dual-luciferase reporter assay. **(A)** Schematic illustration of the two circRNAs and mutant luciferase reporter vectors. **(B, C)** Relative luciferase activities were measured after transfection in EPC with circRNA-WT or circRNA-Mut and miR-200 mimic or miR-NC. *p < 0.05 and **p < 0.01.

To elucidate the underlying molecular mechanism, the miRanda v3.01 toolbox was applied to predict the targeted relationships among circRNA75, circRNA72, and miR-200 in *A. japonicus*. CircRNA75 had four potential binding sites of miR-200, whereas circRNA72 had two potential binding sites. This finding indicated that circRNA75 and circRNA72 may serve as sponges to capture miRNAs, thereby resulting in the release of specific miRNA-targeted transcripts. Wild-type (WT) and mutant (MU) luciferase reporters of circRNA75 and circRNA72 binding sites were constructed to analyze the role of miR-200 in the regulation of the two circRNAs (**Figure 2A**). After co-transfecting EPC cells with miR-200 mimics or NC mimics and the dual-luciferase reporter system, we found that the overexpression of miR-200 significantly inhibited the luciferase reporter activity of four circRNA75 WTs and that of one circRNA72 WT but not the luciferase reporter activity of all circRNA MU (**Figures 2B, C**). All these results validated the binding of miR-200 to circRNA75 and circRNA72.

### Tollip is a Direct Target of MiR-200 to Attenuate LPS-Induced Coelomocyte Apoptosis

**Figure 3:**
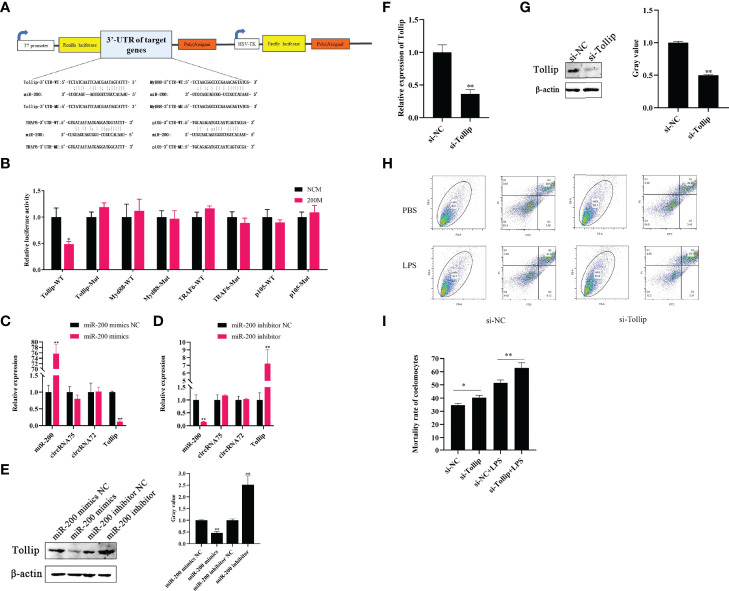
Tollip is a direct target of miR-200 to attenuate LPS-induced coelomocyte apoptosis. **(A)** Schematic illustration of target genes and Mut luciferase reporter vectors. **(B)** Relative luciferase activities were measured in EPC cells after transfection with WT or Mut and a miR-200 mimic or miR-NC. **(C, D)** qRT-PCR was applied to detect the regulation of miR-200 mimics and inhibitors on the mRNA levels of Tollip, circRNA75, and circRNA72. **(E)** Western blot and gray value analysis were used to detect the regulation of miR-200 mimics and inhibitors on the protein levels of Tollip. **(F)** qRT-PCR detected the mRNA level of Tollip after the transfection of si-Tollip. **(G)** Western blot and gray value analyses detected the protein level of Tollip after the transfection of si-Tollip. **(H)** Coelomocyte apoptosis assay after Tollip knockdown *in vitro*. **(I)** Statistical analysis of apoptosis rate after Tollip knockdown. *p < 0.05 and **p < 0.01.

Through the miRanda v3.01 toolbox-screened *A. japonicus* transcriptome data, we predicted the existence of four miR-200 target genes (Tollip, Myd88, TRAF6, and P105). Then, we constructed luciferase reporter plasmids containing the 3’-UTRs of the wild-type (WT) and mutant (MU) genes; the sequences contained miR-200 binding sites (**Figure 3A**). After the dual-luciferase experiment, we found that miR-200 significantly decreased the luciferase activity in the Tollip WT plasmid group but not in other genes and mutant plasmids (**Figure 3B**). Subsequently, qRT-PCR assays revealed that miR-200 mimics significantly reduced Tollip mRNA levels by 0.12-fold and protein levels by 0.46-fold in primary coelomocytes, whereas miR-200 inhibitors increased Tollip mRNA and protein levels by 7.25- and 2.52-fold, respectively (**Figures 3C–E**). Moreover, we found that the expressions of the circRNAs were not affected by the change in miR-200.

Some studies showed that Tollip is an anti-apoptosis gene in higher animals (29). Our previous study proved that Tollip is a negative regulatory gene involved in the activation of the Toll signaling pathway in sea cucumber that showed resistance to *V. splendidus* infection (30). However, the function of Tollip in sea cucumber has not been deeply detected. To further explore the functional role of Tollip, the apoptosis levels of coelomocytes were detected after silencing Tollip *in vitro*. After the si-Tollip was transfected into primary coelomocytes for 24 h, the mRNA and protein levels of Tollip were respectively downregulated by 0.36- and 0.49-fold (*p <*0.01) relative to the levels in the si-NC group (**Figures 3F, G**). Under these conditions, we measured PBS and LPS-induced apoptosis levels by Gallios. The results showed that the mortality rate of the si-Tollip group was upregulated by 4.69 and 9.42% (*p <*0.05) compared with the si-NC group under PBS and LPS-stimulated conditions, respectively (**Figures 3H, I**).

### CircRNA75 and CircRNA72 Could Regulate Tollip-Mediated Coelomocyte Apoptosis by Sponging MiR-200

**Figure 4:**
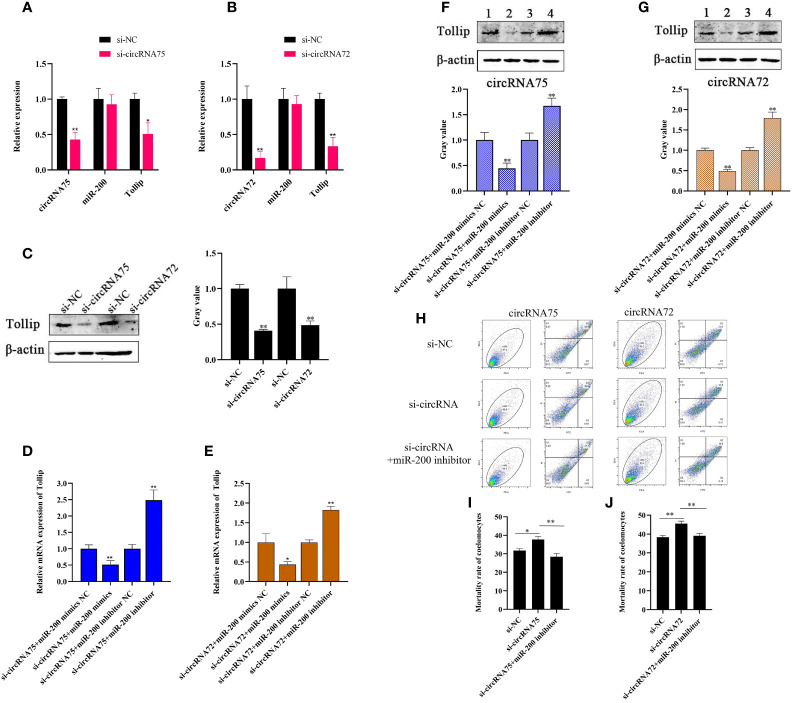
CircRNA75 and circRNA72 could regulate Tollip-mediated coelomocyte apoptosis by sponging miR-200. **(A, B)** After circRNA75 or circRNA72 knockdown, qRT-PCR detected the regulatory effect on Tollip and miR-200 mRNA levels. **(C)** After circRNA75 or circRNA72 knockdown, Western blot and gray value analyses detected the regulatory effects on Tollip protein levels. **(D, E)** Under knockdown conditions of circRNA75 or circRNA72, qRT-PCR detected the effect of miR-200 mimics or inhibitors on the mRNA expression of Tollip. **(F, G)** Under knockdown conditions of circRNA75 or circRNA72, Western blot and gray value analyses detected the effect of miR-200 mimics or inhibitors on the protein expression of Tollip. **(H)** Coelomocyte apoptosis assay after circRNA75 or circRNA72 knockdown and in circRNA knockdown after the addition of the miR-200 inhibitor. **(I, J)** Statistical analysis of apoptosis rate after circRNA75 or circRNA72 knockdown and in the circRNA knockdown of the added miR-200 inhibitor. *p < 0.05 and **p < 0.01.

To investigate whether circRNA75 and circRNA72 participated in the immune response of *A. japonicus* through the sponge activity of miR-200, we designed a specific siRNA that covered the back-splicing region of the two circRNAs for circRNA interference. After the siRNA was transfected, we checked the expressions of circRNAs, miR-200, and Tollip in primary coelomocytes by qRT-PCR and Western blot. The results indicated that si-circRNA75 transfection inhibited circRNA75 expression by 0.43-fold and led to the 0.51- and 0.41-fold decrease in mRNA and protein levels of Tollip, respectively (**Figures 4A, C**). Under the same conditions (si-circRNA75 was replaced by an equivalent amount of si-circRNA72), circRNA72 was depressed by 0.17-fold, which resulted in the 0.33- and 0.49-fold decrease in mRNA and protein levels of Tollip, respectively (**Figures 4B, C**). The circRNA interference replicated the phenomenon of miR-200 overexpression downregulating the expression of Tollip in primary coelomocytes. Meanwhile, the rate of Tollip decrease caused by circRNA75 interference was greater than that caused by circRNA72 interference. However, the expression of miR-200 did not change (**Figures 4A, B**). Furthermore, to functionally confirm that circRNA75 and circRNA72 could serve as sponges of miR-200 to mediate the expression of Tollip, miR-200 mimics and inhibitors were used to examine the change in Tollip expression under circRNA knockdown conditions. The mRNA expression levels of Tollip in si-circRNA75 and si-circRNA72 + miR-200 inhibitor groups were upregulated by 2.49- and 1.83-fold (*p <*0.01) compared with si-circRNA75 and si-circRNA72 + miR-200 inhibitor NC groups, respectively (**Figures 4D, E**), and the protein levels of Tollip were upregulated by 1.67- and 1.80-fold (*p <*0.01) under the same conditions (**Figures 4F, G**). In contrast, mRNA expression levels of Tollip in si-circRNA75 and si-circRNA72 + miR-200 mimics groups were downregulated by 0.51- and 0.43-fold (*p <*0.01) compared with si-circRNA75 and si-circRNA72 + miR-200 mimics NC groups, respectively (**Figures 4D, E**); similarly, the protein expression levels of Tollip were downregulated by 0.44- and 0.49-fold (*p <*0.01; **Figures 4F, G**).

**Figure 5:**
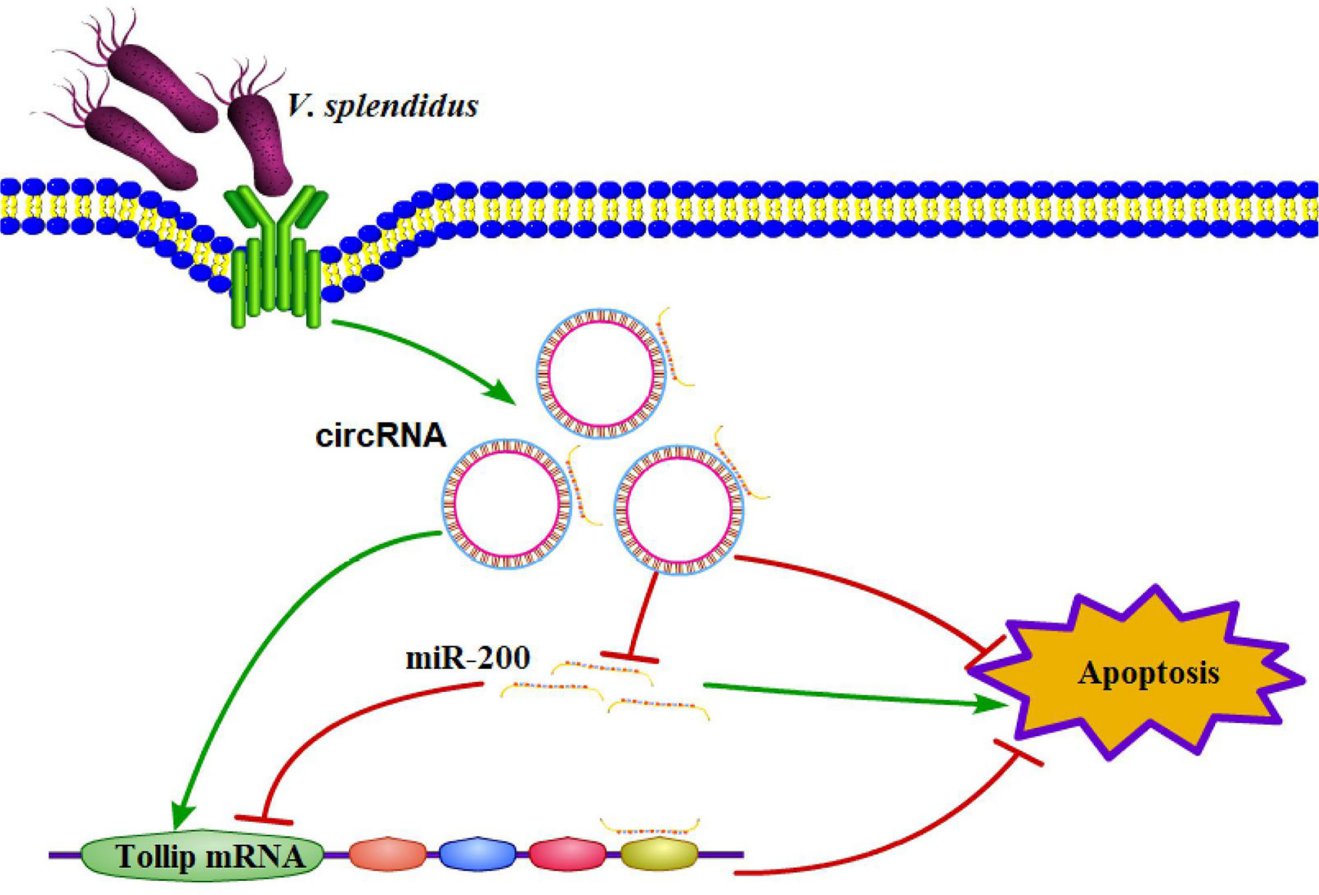
Schematic diagram displaying the mechanism underlying the two circRNAs as endogenous ceRNA for miR-200.

To functionally confirm that circRNA75 and circRNA72 suppressed coelomocyte apoptosis *via* the sponge activity of miR-200 and consequently upregulated Tollip, miR-200 inhibitor was used to examine whether the coelomocyte apoptosis of circRNAs silencing could be blocked by miR-200 knockdown (**Figure 4H**). The results indicated that the mortality rates of the si-circRNA75 and si-circRNA72 groups were significantly upregulated by 5.61 and 6.15%, respectively, compared with that of the si-NC group. However, the mortality rate of si-circRNA75 or si-circRNA72 + miR-200 inhibitor groups showed no change compared with that of the si-NC group (**Figures 4I, J**). All results indicated that circRNA75 and circRNA72, as ceRNA, suppressed cell apoptosis by adsorbing miR-200 and releasing its target Tollip in response to immune stress in *A. japonicus* (**Figure 5**).

## Discussion

High-throughput sequencing illustrated that only a little bit of the whole genome can be translated into proteins, whereas a large proportion of the genome is only transcribed into ncRNAs, which cannot code proteins. CircRNAs have recently been proven to be widespread and highly stable endogenous ncRNAs. CircRNAs are a vital regulator in many biological processes, especially in the occurrence, development, and worsening of human diseases, such as malignant tumors, diabetic retinal vascular dysfunction, fetal growth restriction, and others (31, 32). SUS is a highly contagious and widespread disease that results in great economic loss in the *A. japonic*us culture industry. However, the expression and function of circRNAs, a category of newly discovered RNA, in SUS development are still elusive. In a previous study, 261 circRNAs changed under SUS infection conditions according to the expression profile analysis (26). MiR-200 was considered as an important microRNA related to immunity in *A. japonicus* and other higher organisms (33, 34). Therefore, among the 261 circRNAs, we first screened out three circRNAs (circRNA430, circRNA75, and circRNA72) that were related to miR-200. However, circRNA430 expression in LPS-stimulated coelomocytes displayed inconsistent profiles by qRT-PCR analysis to that from circRNA-seq. Then, we chose circRNA75 and circRNA72 as further study objects. Divergent PCR was used to determine the presence of circRNA75 and circRNA72 in *A. japonicus.* CircRNA75 is derived from one exon and some introns of its host gene fibrinogen-like protein A, which belongs to the fibrinogen-related protein superfamily and is associated with the regeneration of *A. japonicus* (35). CircRNA72 is derived from six exons and some introns of its host gene HSP97, which plays an important role in maintaining cell biological activity, stabilizing cell structure, and repairing cell oxidative damage (36). RNase R is a 3’ to 5’ exonuclease from the RNR superfamily of *Escherichia coli* that can gradually cleave RNA into dinucleotides and trinucleotides from the 3’ to 5’ direction. RNase R can digest almost all linear RNA molecules, but it does not easily digest circRNA (37). When we treated total RNA with RNase R, we found that the two circRNAs were more stable than their host genes and could resist RNase R digestion. In circRNA-seq differential expression analysis, we found that circRNA75 and circRNA72 in the SUS group were upregulated 11.59- and 1.83-fold compared to the healthy group, respectively (26). In the study, the two circRNAs were also upregulated in LPS-stimulated primary coelomocytes, whose expression patterns were similar with those obtained in the circRNA-seq analysis. These results indicated the circRNA75 and circRNA72 exerted an important regulatory effect in response to pathogen infection.

The ceRNA hypothesis stated that RNA transcripts, including mRNAs, circRNAs, lncRNAs, and pseudogene transcripts, can interact with miRNA to control target gene expression (38–41). The RNA transcripts usually competitively adsorb miRNA response elements (MREs) with mRNA, thereby building a complex posttranscriptional regulatory network. More and more researchers demonstrated that circRNAs exhibit miRNA sponge activity, which was a key factor in the occurrence of many diseases and the most important action mechanism of circRNA (41). In this study, we confirmed by dual-luciferase assay that circRNA75 and circRNA72 had a high binding capacity with miR-200 *in vivo*. In addition, the knockdown of circRNA75 and circRNA72 could not modulate miR-200 expression in primary coelomocytes. The result indicated that circRNA75 and circRNA72 served as miRNA sponges of miR-200. As competing endogenous RNAs, circRNA75 and circRNA72 were not able to regulate the total expression of miR-200. They only affected the unbound form of miR-200.

Some studies indicated that miRNAs could participate in the degradation of target mRNA, leading to the inhibition of target mRNA translation (42, 43). In the study, we predicted the presence of four potential target genes (Tollip, Myd88, TRAF6, and P105) through the miRanda v3.01 toolbox-screened *A. japonicus* transcriptome data. Furthermore, results of the dual-luciferase assay showed that 3’-UTR of Tollip had direct binding sites for miR-200. The mRNA levels of Tollip were downregulated after miR-200 overexpression. When miR-200 was knocked down, the mRNA levels of Tollip were significantly upregulated. Therefore, these results illustrated that Tollip was a direct target gene for miR-200. Tollip has been reported to be a member of the Toll-like receptor (TLR) signaling pathway and considered as an important negative regulator in the pathogenic infection of *A. japonicus* (30, 44). In our previous study, we found Tollip could modulate the antibacterial activities and suppress LPS priming capacity in *A. japonicus* coelomocytes (45). However, the function of Tollip in *A. japonicus* has not been deeply detected. Some studies indicated that Tollip could exert anti-apoptosis and pro-autophagy effects (29). Tollip protects intestinal epithelial cells from apoptosis induced by interferon gamma and tumor necrosis factor alpha signaling (46). In idiopathic pulmonary fibrosis, the global downregulation of the Tollip gene could predispose injured lung epithelial cells to apoptosis and to the development of idiopathic pulmonary fibrosis (47). The apoptosis function of Tollip was consistent with some reported circRNAs (48, 49). In this study, we also found a similar phenomenon, i.e., Tollip knockdown could resist LPS-induced apoptosis of primary coelomocytes.

Tollip is a direct target gene for miR-200 and has an anti-apoptosis function. Therefore, whether circRNA75 and circRNA72, which serve as miR-200 sponges, could mediate apoptosis of primary coelomocytes through miR-200/Tollip is unknown. We discovered that attenuated circRNA75 and circRNA72 expressions could decrease Tollip expression and promote apoptosis of primary coelomocytes. We also found that the rate of si-circRNA75’s regulation of Tollip was higher than that of si-circRNA72 under an equivalent amount, which demonstrated that the rate of Tollip decrease might be positively related to the number of binding sites for miRNA. Furthermore, under circRNA75 and circRNA72 silencing conditions, adding the miR-200 inhibitor could upregulate the Tollip mRNA levels and abolish the apoptosis-promoting effect of circRNA75 and circRNA72 silencing. All these data convincingly demonstrated that circRNA75 and cricRNA72 served as a sponge of miR-200 and suppressed apoptosis through the circRNAs/miR-200/Tollip axis.

In summary, our studies revealed that circRNA75 and circRNA72 expressions were significantly upregulated in LPS-stimulated primary coelomocytes. Functionally and mechanistically, circRNA75 and circRNA72 inhibited apoptosis of primary coelomocytes through the sponge activity on miR-200 and upregulation of Tollip expression. The ability of circRNA to adsorb miR-200 might be positively related to the number of binding sites for miR-200. Our data suggest that circRNA75 and cricRNA72 may have great potential as prognosis predictors and therapeutic targets for SUS. The regulatory network involving the circRNA/miR-200/Tollip axis might provide new ideas for the targeted treatment of SUS.

## Data Availability Statement

The raw data supporting the conclusions of this article will be made available by the authors, without undue reservation.

## Ethics Statement

The experimental protocols were approved by the Research Animal Ethics Committee of Ningbo University, China.

## Author Contributions

JL, CL, and XZ conceived and designed the experiments and wrote the manuscript. JL and XD conducted the experiments. JL and XZ analyzed the data. CL and WZ contributed to the reagents, materials, and analysis tools. All authors contributed to the article and approved the submitted version.

## Funding

This work was supported by the National Key R&D Program of China (2018YFD0900305), the National Natural Science Foundation of China (32073003, 31702376), the Natural Science Foundation of Zhejiang Province (LZ19C190001, LY21C190005), the Natural Science Foundation of Ningbo (2018A610345), and the K.C. Wong Magna Fund in Ningbo University.

## Conflict of Interest

The authors declare that the research was conducted in the absence of any commercial or financial relationships that could be construed as a potential conflict of interest.

## Publisher’s Note

All claims expressed in this article are solely those of the authors and do not necessarily represent those of their affiliated organizations, or those of the publisher, the editors and the reviewers. Any product that may be evaluated in this article, or claim that may be made by its manufacturer, is not guaranteed or endorsed by the publisher.
