# Corrigendum: CircRNA75 and CircRNA72 Function as the Sponge of MicroRNA-200 to Suppress Coelomocyte Apoptosis *Via* Targeting Tollip in *Apostichopus japonicus*

**DOI:** 10.3389/fimmu.2022.892511

**Published:** 2022-05-17

**Authors:** Jiqing Liu, Xuelin Zhao, Xuemei Duan, Weiwei Zhang, Chenghua Li

**Affiliations:** ^1^ State Key Laboratory for Quality and Safety of Agro-products, Ningbo University, Ningbo, China; ^2^ Collaborative Innovation Center for Zhejiang Marine High-efficiency and Healthy Aquaculture, Ningbo University, Ningbo, China; ^3^ Laboratory for Marine Fisheries Science and Food Production Processes, Qingdao National Laboratory for Marine Science and Technology, Qingdao, China

**Keywords:** *Apostichopus japonicus*, circRNA, miR-200, Tollip, apoptosis

In the original article, there was a mistake in **Figure 3E** as published. In **Figure 3E**, the label of the western blot should be miR-200; however, we inadvertently it replaced with miR-2008. Therefore, we accidentally wrote miR-200 as miR-2008 due to our carelessness. The images of **Figure 3E** should be changed. The corrected **Figure 3** appears below.

**Figure 3:**
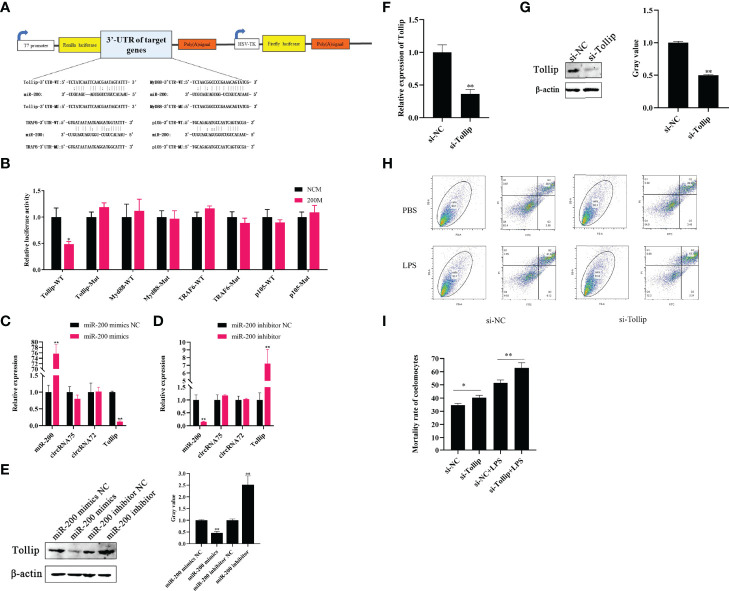
Tollip is a direct target of miR-200 to attenuate LPS-induced coelomocyte apoptosis. **(A)** Schematic illustration of target genes and Mut luciferase reporter vectors. **(B)** Relative luciferase activities were measured in EPC cells after transfection with WT or Mut and a miR-200 mimic or miR-NC. **(C, D)** qRT-PCR was applied to detect the regulation of miR-200 mimics and inhibitors on the mRNA levels of Tollip, circRNA75, and circRNA72. **(E)** Western blot and gray value analysis were used to detect the regulation of miR-200 mimics and inhibitors on the protein levels of Tollip. **(F)** qRT-PCR detected the mRNA level of Tollip after the transfection of si-Tollip. **(G)** Western blot and gray value analyses detected the protein level of Tollip after the transfection of si-Tollip. **(H)** Coelomocyte apoptosis assay after Tollip knockdown in vitro. **(I)** Statistical analysis of apoptosis rate after Tollip knockdown. *p < 0.05 and **p < 0.01.

The authors apologize for this error and state that this does not change the scientific conclusions of the article in any way. The original article has been updated.

## Publisher’s Note

All claims expressed in this article are solely those of the authors and do not necessarily represent those of their affiliated organizations, or those of the publisher, the editors and the reviewers. Any product that may be evaluated in this article, or claim that may be made by its manufacturer, is not guaranteed or endorsed by the publisher.

